# TRIM32-Cytoplasmic-Body Formation Is an ATP-Consuming Process Stimulated by HSP70 in Cells

**DOI:** 10.1371/journal.pone.0169436

**Published:** 2017-01-04

**Authors:** Yuki Kawaguchi, Masato Taoka, Takahiro Takekiyo, Takamasa Uekita, Ikuo Shoji, Naomi Hachiya, Tohru Ichimura

**Affiliations:** 1 Department of Applied Chemistry, National Defense Academy, Yokosuka, Kanagawa, Japan; 2 Department of Chemistry, Tokyo Metropolitan University, Hachioji, Tokyo, Japan; 3 Division of Infectious Disease Control, Center for Infectious Diseases, Kobe University Graduate School of Medicine, Kobe, Japan; 4 Biotechnology Group, R&D Division, Tokyo Metropolitan Industrial Technology Research Institute, Koto-ku, Tokyo, Japan; Boston University Medical School, UNITED STATES

## Abstract

The spontaneous and energy-releasing reaction of protein aggregation is typically prevented by cellular quality control machinery (QC). TRIM32 is a member of the TRIM (tripartite motif-containing) ubiquitin E3 ligases, and when overexpressed in cultured cells, readily forms spherical inclusions designated as cytoplasmic bodies (CBs) even without proteasome inhibition. Here, we show that HSP70, a central QC component, is a primary binding factor of overexpressed TRIM32. Contrary to expectation, however, we find that this molecular chaperone facilitates and stabilizes CB assembly depending on intrinsic ATPase activity, rather than preventing CB formation. We also show that the HSP70-TRIM32 complex is biochemically distinct from the previously characterized 14-3-3-TRIM32 phospho-complex. Moreover, the two complexes have opposing roles, with HSP70 stimulating CB formation and 14-3-3 retaining TRIM32 in a diffuse form throughout the cytosol. Our results suggest that CB inclusion formation is actively controlled by cellular QC and requires ATP, similar to protein folding and degradation reactions.

## Introduction

Although protein aggregation occurs spontaneously through a decrease of free energy [1], this reaction is typically prevented in cells by the protein quality control machinery (QC). The QC system has at least two means of deterring protein aggregation: 1) molecular chaperones facilitate the folding or refolding of aberrantly folded proteins; or 2) ubiquitin-proteasome and autophagy systems remove such proteins through proteolytic degradation [2–4]. The accumulation of protein aggregates inside cells has nonetheless been documented under certain circumstances, including diseases and aging. Such accumulation is thought to occur through the excessive generation of misfolded molecules that surpasses the refolding or degradative capacity of cellular QC [2,3].

**Fig 1:**
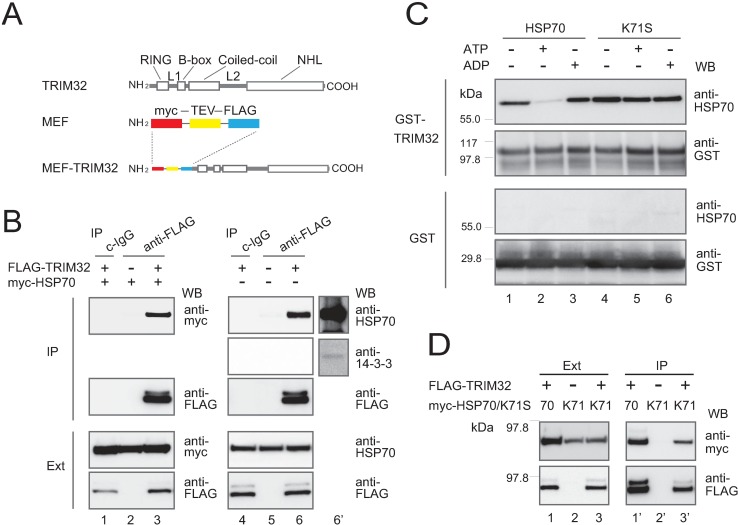
Identification of HSP70 as a major binding partner of the overexpressed TRIM32 polypeptide. (**A**) Schematic representation of the human TRIM32 protein (upper) and its MEF-fused version (lower). (**B**) Association of TRIM32 and HSP70 as assessed with western blotting (WB). HEK293 cells were transfected with the FLAG-TRIM32 construct or the vector alone, with (lanes 1–3) or without (lanes 4–6) myc-HSP70. The lysates were separated with SDS-PAGE and western-blotted with anti-FLAG (for FLAG-TRIM32), anti-myc (for myc-HSP70), or anti-HSP70 (for endogenous HSP70) (lower panels, marked as “Ext”). FLAG-TRIM32 proteins were also immunoprecipitated from the lysates with nonimmune IgG (c-IgG) or anti-FLAG Sepharose beads (anti-FLAG). The immunoprecipitates were western-blotted with the aforesaid antibodies (upper panels, marked as “IP”). Lane 6′ represents lane 6 at a higher exposure. (**C**) HSP70 directly binds to TRIM32 depending on the specific nucleotide-binding state. Purified GST-TRIM32 or GST alone immobilized on agarose beads was incubated with purified HSP70 (lanes 1–3) or the K71S mutant protein (lanes 4–6) in the presence or absence of ATP or ADP, and then western-blotted with anti-HSP70 (for HSP70 and K71S) or anti-GST (for GST-TRIM32 and GST). “+” and “-” respectively indicate presence and absence of ATP/ADP. (**D**) FLAG-TRIM32-myc-HSP70/K71S-transfected HEK293 cells were co-immunoprecipitated to analyze the association between TRMI32 and K71S, as described in (B).

Tripartite motif-containing protein 32 (TRIM32) is a member of the TRIM ubiquitin E3 ligases. This protein family is characterized by the RING finger, B-box, and coiled-coil domains; TRIM32 also possesses a unique NHL domain at the C-terminus (see Fig 1A). Like many other TRIM E3 ligases, TRIM32 has a strong tendency to aggregate. Indeed, when overexpressed in cultured human and mouse cells, TRIM32 readily forms spherical hollow inclusions designated as cytoplasmic bodies (CBs) despite intact proteasome activity [5–8]. In general, when TRIM proteins (including TRIM32) are expressed at low levels, they are distributed diffusely throughout the cytoplasm predominantly as small foci or dots. As expression increases, the small TRIM structures also increase in size and fuse to form larger cytoplasmic clusters, so-called CBs [5,8,9].

We currently know little about the physiological role of CBs in relation to TRIM32 function. However, previous studies, particularly with TRIM5α and TRIM50, have suggested that CBs might act as aggresome precursors or storage compartments that sequester overexpressed, potentially misfolded or partially folded TRIM molecules, allowing for their eventual refolding or degradation [9–12]. Controlling cellular TRIM32 concentration may be critical for healthy cell physiology because abnormally high concentrations have been demonstrated in human skin cancer cells [13] and the occipital lobe of Alzheimer’s disease patients [14]. It has also been shown that severe reduction of TRIM32 levels causes several myopathic and neuropathological defects and increases the susceptibility of tracheal and lung epithelial cells to influenza A-virus infection [15–17]. This potential role of CBs suggests that the QC system might actively regulate their formation, but the possibility has not been fully explored. No information on the cellular machinery driving CB assembly has been reported, except that small TRIM-containing bodies move along microtubules [8,9,18].

In this work, we confirm previous studies by showing that the central QC component, HSP70, is indeed a primary binding partner of TRIM32 when the ligase is overexpressed in HEK293 cells. Contrary to expectation, however, we also find that HSP70 actually enhances and possibly stabilizes CB formation, rather than inhibiting the process. The activity of HSP70 was dependent on intrinsic ATPase activity. Our findings suggest that CB inclusion formation is like protein folding and degradation; all three are ATP-requiring reactions that cellular QC actively controls.

## Materials and Methods

### Materials

The mammalian expression plasmid carrying MEF (myc-TEV-FLAG)-tagged TRIM32 (MEF-TRIM32) was generated with PCR, using oligonucleotides 5′-CGGAATTCATGGCTGCAGCAGCAGCTTC-3′ and 5′-CGGAATTCCTATGGGGTGGAATATCTTC-3′, as well as human TRIM32 cDNA (pOTB7, Funakosi) as a template. The PCR fragment was digested with *Eco*RI and then inserted into the pcDNA3-MEF vector [19]. The expression plasmids of FLAG-TRIM32, myc-14-3-3η and the catalytic subunit of PKA have been described previously [8].

To create myc-HSP70 and myc-HSP90 expression constructs, human HSP70 (HSPA1A [20]) and HSP90 (HSP90AA1 [21]) cDNAs were digested with *Bam*HI and *Eco*RI, then inserted into pCMV-Tag3B (Invitrogen). The point mutants of HSP70 (K71S and V438F) and HSP90 (D88N) were created using PCR-based targeted mutagenesis and confirmed with DNA sequence analysis. The expression plasmids for HSP40 (HDJ1; [22]) and FLAG-TRIM63 [23] were provided by Drs. Ohtsuka (Gifu University) and Inoue (University of Tokyo), respectively. Small interfering RNA (siRNA) targeting HSP70 (HSPA1A) was purchased from Dharmanon. Control siRNA was from Santa Cruz Biotechnology.

To produce the GST-fused TRIM32, HSP70, and K71S, the GST portion of pGEX-3X (GE Healthcare Life Sciences) was PCR-amplified and inserted into the bacterial expression plasmid pET22b (hereafter, pET-GST; Novagen). The pET-GST plasmid was digested with *Bam*HI and *Eco*RI and then inserted with TRIM32, HSP70, and K71S, obtained through digestion of FLAG-TRIM32, myc-HSP70, and myc-K71S using the same restriction enzymes. Protein expression and affinity purification with glutathione Sepharose beads were performed as previously described [24] except for the incubation for 4 h at 25°C and the use of 0.15 mM IPTG (isopropyl β-D-1-thiogalactopyranoside). The purified GST-HSP70 and GST-K71S proteins were then cleaved with Factor X (Bio-Rad) to obtain HSP70 and K71S. Monoclonal anti-myc and anti-FLAG were obtained from Santa Cruz Biotechnology and Kodak, respectively. Polyclonal anti-myc and anti-FLAG antibodies were purchased from Cell Signaling and SIGMA, respectively. Monoclonal anti-HSP70 was purchased from BD Biosciences or StressMarq Biosciences for the experiments shown in S4 Fig. The PAN-14-3-3 antibodies (K-19) were from Santa Cruz Biotechnology. MB, AlaC, and SW02 were obtained from WAKO, Fluka, and SIGMA, respectively.

### MEF purification and mass spectrometry

Five 10-cm dishes of HEK293 cells were transiently transfected with the MEF-TRIM32 plasmid (5 μg/dish). After 24 h, the cells were lysed in 2.5mL of lysis buffer (50mM Tris-HCl, pH7.5, 150mM NaCl, 10% [w/v] glycerol, 100mM NaF, 10mM EGTA, 1mM Na_3_VO_4_, 1% [w/v] Triton X-100, 5mM ZnCl_2_, 2mM phenylmethylsulfonyl fluoride, 10μg/mL aprotinin, and 1μg/mL leupeptin). Expressed MEF-TRIM32 and bound proteins were recovered following the MEF method [19]: the lysate was first immunoprecipitated with anti-myc-conjugated Sepharose beads and cleaved from the beads using TEV protease. Next, the dissociated proteins were immunoprecipitated again with anti-FLAG-Sepharose beads, and finally eluted from the FLAG beads with synthetic FLAG peptides (80 μg/mL; SIGMA). The eluted proteins were digested with trypsin and then identified using nanoACQUITY UPLC–Xevo G2-S QTof system (Waters). Accurate LC-MS data were collected using the data-independent MSe procedure [25]. The LC-MSe data were processed using TransOmics Informatics (Waters) and searched against the human protein database from UniProt (http://uniprot.org/).

### Co-immunoprecipitation and pull-down assay

Co-immunoprecipitation analyses were conducted as described previously [8,19]. We transiently transfected HEK293 cells (2–4 × 10^6^) with FLAG-TRIM32 expression plasmids that were either with or without myc-HSP70 or K71S (5μg each). After 24 h, the expressed FLAG–TRIM32 was immunoprecipitated with anti-FLAG–Sepharose (SIGMA), washed five times with buffer A (50mM Tris-HCl, pH7.5, 150mM NaCl, 10% [w/v] glycerol and 0.1% [w/v] Triton X-100), and dissociated from the beads with 80 μg/mL synthetic FLAG peptide. Proteins were then separated using 10% or 7.5% SDS-PAGE and western-blotted with specific antibodies indicated in each figure.

For GST pull-down experiments, 10μg of HSP70 or K71S was pre-incubated for 10 min at room temperature in a 50-μL reaction volume containing 20mM Tris-HCl (pH7.5) and 3mM MgCl_2_. Additionally, 2 mM ATP or ADP was either present or absent depending on the experimental condition. After incubation, GST-TRIM32 (0.5μg) absorbed on glutathione-Sepharose beads (GE Healthcare) were added to the reaction mixture, which was incubated for another 60min at room temperature. The beads were washed four times with buffer A, and bound proteins were again analyzed with 7.5% SDS-PAGE, followed by western blotting.

### Immunofluorescence microscopy

We performed immunofluorescence microscopy following previously described methods [8]. First, HEK293 cells grown on 3.5-cm plates (FluoroDish, World Precision Instruments, Inc.) were transfected with 0.5 μg of expression plasmids for 24 h using Lipofectamine 2000 (Invitrogen). Transfected cells were then rinsed with PBS(+) (phosphate-buffered saline containing 0.1 g/L CaCl_2_ and 0.1 g/L MgCl_2_) and fixed with 3.7% formalin in 70% PBS(+) for 30 min at 25°C. The fixed cells were washed four times with PBS, and then incubated with 10% fetal bovine serum in PBS for 30 min at room temperature. Subsequently, the cells were incubated for another 1 h at room temperature with monoclonal mouse anti-FLAG and polyclonal rabbit anti-myc. Next, the cells were washed four times with PBS again; then, they were incubated with Alexa-Fluor-488-conjugated (green) anti-mouse and Alexa-Fluor-594-conjugated (red) anti-rabbit secondary antibodies (Molecular Probes) for 1 h at room temperature. Finally, the stained cells were washed another four times with PBS and then visualized using a DeltaVision microscope system (Applied Precision, Inc.). Analysis using three-dimensional image reconstitution was performed in SoftWoRx (Applied Precision, Inc.). For the experiments shown in S2 Fig and S4 Fig, the fluorescence images were taken using an Olympus FluoView FV10i microscope. We defined CBs as spherical, hollow, and >0.3 μm particles observed under microscopy and characterized them as described in the results.

### MB treatment

HEK293 cells were transfected with the FLAG-TRIM32 plasmid. After 24 h, 50 μM MB and 100 μg/mL CHX (both dissolved in DMSO) were added to transfected cells. Culturing then continued for 2 h and 4 h.

### Statistical analysis

Statistical analyses of CB size and volume, as well as CB-containing cell number, were performed in Microsoft Excel. Graphical plots were also made in Excel. Data were expressed as means ± SD. The number of cells analyzed is indicated in the figure legends.

## Results and Discussion

### Identification and confirmation of HSP70 as a primary binding partner of overexpressed TRIM32

To identify proteins that control the intracellular localization of TRIM32, we used a MEF (myc-TEV-FLAG) tag cassette [19, 26]. The MEF cassette was fused to the N terminus of human TRIM32 (Fig 1A), and the MEF-fused version of TRIM32 was transiently overexpressed in HEK293 cells under normal culture conditions. The interacting partners that associated specifically with MEF-TRIM32 were then extracted from the cell lysate, using MEF-based multi-step immunoaffinity purification [19], and analyzed by mass spectrometry.

We were able to identify several known and unknown TRIM32 targets, including 14-3-3 proteins, consistent with our previous analysis, even in the absence of protein kinase A (PKA) activator [8]. We found, however, that many other targets were more abundant in identified peptide number and sequence coverage than 14-3-3s under these conditions, including the following QC components: the chaperone HSP70 (HSPA1A) and its family members, several subunits of the chaperonin TRiC, and polyubiquitin B. In particular, HSP70 is a molecular chaperone that is central to the QC system, so we further analyzed this interaction through western blots with antibodies specific to ectopic myc-HSPA1A or endogenous HSP70. Both overexpressed myc-HSPA1A and endogenous HSP70 were more abundantly detected in TRIM32 immunoprecipitates (Fig 1B, lanes 3 and 6) than endogenous 14-3-3s (Fig 1B, lanes 6 and 6’), but not detected at all in control immunoprecipitates (Fig 1B, lanes 1–2 and 4–5). We also confirmed that the two antibodies used for the analysis of endogenous interactions (anti-HSP70 and anti-14-3-3) similarly and effectively bind to the purified proteins [detection limit, ~0.5 ng per HSPA1A and bovine 14-3-3η spot (0.07 ng/mm^2^)] in a dot-immunobinding assay on a PVDF (polyvinylidene difluoride) membrane. Thus, these results show that HSP70 is a primary binding target of overexpressed TRIM32 in HEK293 cells.

### HSP70 interacts with TRIM32 in a classical ATP-regulated reaction cycle

Protein folding chaperoned by HSP70 involves ATP-regulated reaction cycles of substrate binding and release [27]. Specifically, ATP-bound HSP70 shows low affinity for protein substrates, but forms strong bonds once ATP is hydrolyzed or if it is nucleotide-free. To investigate whether the HSP70-TRIM32 interaction is similar to previous descriptions, we overexpressed a GST-fused TRIM32, and the expressed fusion immobilized on glutathione–Sepharose beads was assayed for HSP70-binding under ATP/ADP presence or absence. This analysis clearly demonstrated the direct binding of HSP70 to immobilized GST-TRIM32 (Fig 1C, upper 2 panels, lanes 1–3). In contrast, no or less association was detected with the GST moiety alone, confirming that the binding occurred specifically with the TRIM32 portion (Fig 1C, lower 2 panels, lanes 1–3). Furthermore, the interaction was much stronger (~10-fold) in the ADP-bound or nucleotide-free states than in the ATP-bound state, as estimated by densitometric quantitation of the corresponding bands (Fig 1C, top panel, lanes 1–3).

We then produced the dominant-negative HSP70 K71S variant (an HSP70 ATP-binding-site mutant) to further characterize the interaction. This variant exhibits wild-type HSP70 behavior in an ADP-bound, high-substrate-affinity conformation [28], binding to TRIM32 in vitro (Fig 1C, lanes 4–6) and in cells (Fig 1D, lane 3’). However, this mutation abolishes intrinsic ATPase potential and chaperone activity, and fixes HSP70 only in a high substrate affinity state [28,29]. Our results showed that the K71S mutant indeed bound to TRIM32 even in the presence of ATP (Fig 1C, top panel, lane 5). Thus, the interaction with TRIM32 conforms to the reported characteristics of HSP70-substrate binding. These results provide evidence suggesting that TRIM32 is a substrate of HSP70 chaperone, and that their interaction is under dynamic control by the specific nucleotide-binding state of HSP70.

### HSP70 facilitates the formation of TRIM32-containing CBs depending on intrinsic ATPase activity

Confirming previous reports [8], our FLAG-tagged TRIM32 construct readily formed CBs when overexpressed in HEK293 cells. These TRIM32-containing CBs exhibited near-spherical, hollow morphology (average size and volume: 0.60 ± 0.08 μm and 0.12 ± 0.04 × 10 μm^3^, respectively; mean ± SD), and were generally detected in ~60% of total TRIM32-positive cells 24 h post-transfection, either in peripheral or juxtanuclear locations (S1A Fig; S1 Movie). The TRIM32 CBs were not stained with anti-ubiquitin antibody (FK2) and not co-localized with vimentin, as would be expected for aggresomes. Importantly, when the transfected cells were treated with protein synthesis inhibitor cycloheximide (CHX, 100 μg/mL), almost all visible CBs disappeared within 18 h. Quantitative immunoblotting revealed that the apparent half-life (t_1/2_) of TRIM32 was ~8 h (S1B Fig). Together, the overexpression data and the CHX data suggest that TRIM32-containing CBs are dynamic compartments in living cells where newly synthesized TRIM32 molecules constantly accumulate and undergo degradation. Moreover, neither proteasome nor autophagy inhibitors (MG132 [10 μM] and clasto-Lactacystin [10 μM], or NH_4_Cl [50 mM]) prevented TRIM32 degradation, suggesting that pathway(s) distinct from typical protein degradation processes could mediate this turnover. Similar resistance to proteasome and autophagy inhibitors has been reported for the turnover of TRIM5α protein stably expressed in HeLa or Cf2Th cells [11,30].

**Fig 2:**
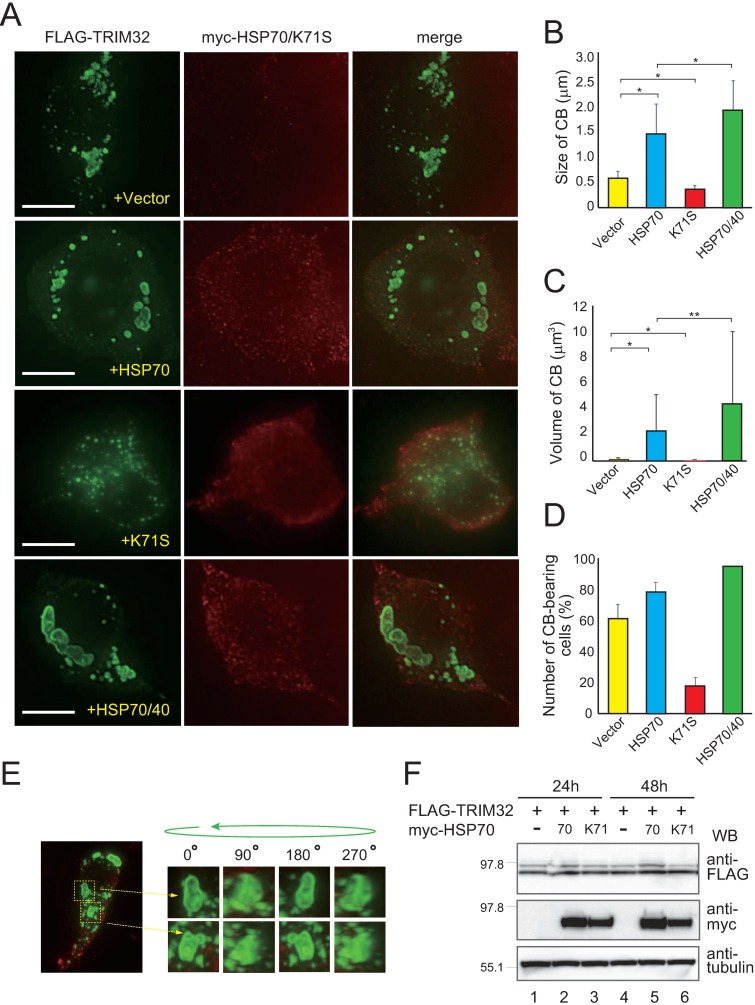
HSP70 promotes the formation of TRIM32-containing CBs depending on ATPase activity. (**A**) HEK293 cells were transfected with FLAG-TRIM32 and the constructs (names indicated in yellow) were fixed and stained with monoclonal mouse anti-FLAG (green) and polyclonal rabbit anti-myc (red). Representative images of cells from at least three independent experiments are shown. Bars, 10 μm. (**B–D**) HEK293 cells were stained as described in (A); the size (B) and volume (C) of TRIM32-containing CBs, as well as the number of CB-containing HEK293 cells (D) were quantified from 100–500 cells in at least three experimental repeats. Volumes were determined with the assumption that CBs are round. Data are means ± SD. * indicates P < 0.0001 and ** indicates P < 0.0005. (**E**) Three-dimensional image of TRIM32 CBs in myc-HSP70-expressing HEK293 cells. (**F**) Whole proteins from HEK293 cells were extracted with 1% SDS after 24 h (lanes 1–3) or 48 h (lanes 4–6) of transfection with HSP70 or K71S plasmids, and analyzed with western blotting. Tubulin was used as an internal control.

We then co-transfected HEK293 cells with myc-HSP70 or empty vector, and used microscopy to examine how HSP70 affected the assembly of TRIM32-containing CBs. Unexpectedly, we found that HSP70 clearly accelerated both the rates of generation and the growth of TRIM32 CBs, rather than inhibiting these processes (Fig 2A, second set of panels from the top), whereas vector alone had no effect. Indeed, the CBs in HSP70-expressing cells were routinely larger and rounder (~3-fold increase in size from empty vector, p < 0.0001; ~20 fold increase in volume from empty vector, p < 0.0001; Fig 2B and 2C). Additionally, 80% of HSP70-expressing cells contained CBs, compared with 60% of the vector-alone cells (Fig 2D). Three-dimensional image reconstitution analysis further indicated that a fraction of the myc-HSP70-expressing cells contained crowded CB clusters that may continue coalescing into larger CBs (Fig 2E; S2 Movie). While the GST pull-down assay described above demonstrated that HSP70 binds directly to TRIM32 (Fig 1C), it is uncertain whether this binding was actually involved in the observed CB assembly pathway. To address this issue, we tested the effect of HSP70 V438F mutant. This mutant has a point mutation in the substrate-binding pocket of human HSP70 [31, 32] and therefore is unable to associate with TRIM32 when expressed in HEK293 cells (S2A Fig). Analysis of the effect of V438F expression on CB formation revealed that this mutant did not show any obvious CB growth phenotypes observed with wild-type HSP70 (S2B Fig), supporting the notion that HSP70 binding is important for the CB formation.

Next, we determined whether the intrinsic ATPase activity of HSP70 was associated with the observed CB changes. To this end, we again employed the ATPase-deficient K71S variant. In sharp contrast to wild-type HSP70, the co-expressed K71S variant significantly decreased CB size and volume by 0.6-fold (p < 0.0001; Fig 2B) and 0.2-fold (p < 0.0001; Fig 2C), respectively. Moreover, less than 20% of K71S-expressing cells had CBs (Fig 2D), instead possessing numerous and small (<0.2 μm) TRIM32-containing structures that were often distributed throughout the cytoplasm (Fig 2A, third set of panels from the top; we did not count these dot-like structures as CBs). This defect in CB assembly was not due to a reduction in total TRIM32 levels, because comparative immunoblotting revealed that K71S-transfected and non-transfected cell lysates exhibited near-equal amounts of TRIM32, even after 48 h (Fig 2F, top panel). These results suggest that TRIM32 CB formation is an active process rather than a spontaneous and uncontrolled pathway. Specifically, the active reaction of CB formation is stimulated by the HSP70 chaperone in a manner dependent on intrinsic ATPase activity. As further support, we found that when HSP70 ATPase activity was activated by the co-expression of co-chaperone HSP40 (HDJ1), CB formation was accelerated (Fig 2A–2D, HSP70/40).

To understand whether the observed effects occurred with other HSP family members, we used myc-HSP90 (HSP90AA1) and its ATPase-dead D88N mutant [33]. Both of these molecules bound to FLAG-TRIM32 when overexpressed in HEK293 cells, but they did not affect the basal CB-assembly pathway (S3 Fig). Thus, the role of HSP70 chaperone on CB formation appears to be specific and biologically important.

### Functional HSP70 chaperone is required for TRIM32-containing CBs to maintain structural integrity in the cell

**Fig 3:**
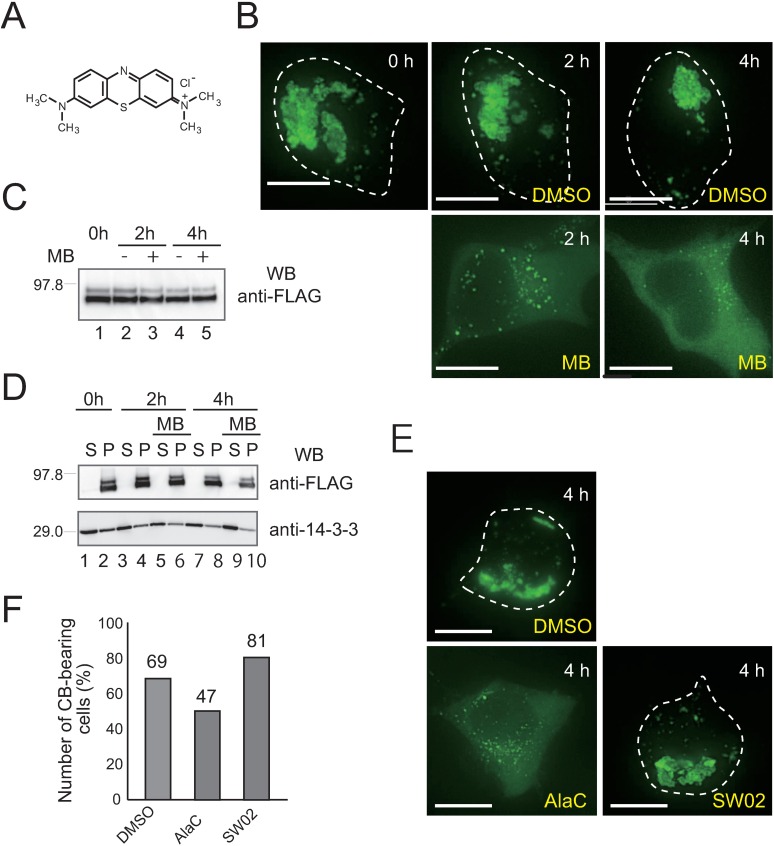
Methylene blue (MB) disrupts the structure of TRIM32-containing CBs in HEK293 cells. (**A**) MB chemical structure. (**B**) Fluorescence microscopy images of HEK293 cells with preformed TRIM32 CBs that were exposed to either DMSO or 50 μM MB dissolved in DMSO for 2 and 4 h. (**C**) FLAG-TRIM32-transfected HEK293 cells with preformed TRIM32 CBs were exposed to 50 μM MB dissolved in DMSO (+) or DMSO only (-) for 2 and 4 h. Whole proteins were extracted with 1% SDS and western-blotted with anti-FLAG to monitor total TRIM32 levels. (**D**) HEK293 cells with preformed TRIM32 CBs were treated with DMSO and MB, then homogenized in PBS. Western blots of soluble (S) and insoluble (P) proteins with anti-FLAG (upper panel) and anti-PAN 14-3-3 antibodies (lower panel). The cytosolic marker 14-3-3 was used as a loading control for soluble and insoluble fractions. (**E**) Fluorescence microscopy images of HEK293 cells with preformed TRIM32 CBs that were exposed to DMSO, AlaC (50 μM), or SW02 (50 μM) for 4 h. (**F**) The percentage (out of ~400 cells) of CB-containing HEK293 cells exposed to DMSO, AlaC or SW02. Bars in (B) and (E), 10 μm.

**Fig 4:**
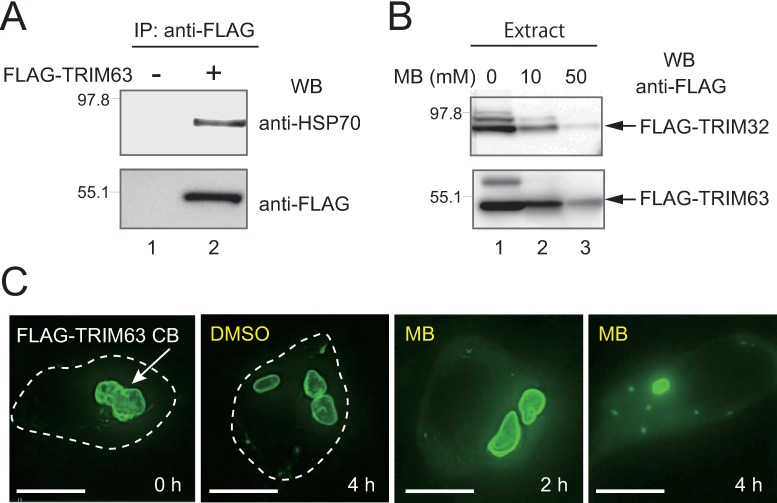
HSP70 stabilizes TRIM63-containing CBs in HEK293 cells. (**A**) HEK293 cells were transiently transfected with FLAG-TRIM63 (+) or vector alone (-). The association of TRIM63 with endogenous HSP70 was analyzed with co-immunoprecipitation followed by western blotting (WB). (**B**) HEK293 cells transfected with FLAG-TRIM32 (upper panels) or FLAG-TRIM63 (lower panels) were incubated for 24 h in the presence or absence of MB. Total TRIM32/63 levels were analyzed with western blotting. (**C**) Fluorescence microscopy images of HEK293 cells with preformed TRIM63-containing CBs, exposed to either DMSO or MB for 2 or 4 h. Bars in (C), 10 μm.

Methylene blue (MB; Fig 3A) is a benzothiazine compound under clinical investigation for treatments of neurological diseases, including Alzheimer’s and Huntington's, because it is capable of reducing tau and huntingtin aggregate levels in several cell and animal models [34,35]. Based on a recent report of MB acting as a specific inhibitor of HSP70 ATPase activity [36], we examined whether MB affects the CB assembly pathway. However, similar to its effect on Akt kinase and tau [36,37], MB significantly reduced total TRIM32 levels through an unknown mechanism when added at the time of transfection with the FLAG-TRIM32 plasmid (see Fig 4B, upper panel). Thus, initial trials to monitor MB effects on CB assembly were unsuccessful.

Therefore, we first stimulated CB formation in HEK293 via transfection with the FLAG-TRIM32 plasmid. We then subjected these cells to either an MB or a no-MB treatment in the presence of CHX. Because incubation with CHX over 6 h induced detachment of almost all MB-treated HEK293 cells from glass-bottom dishes, a relatively short incubation period (2 h and 4 h) was also employed for these experiments. We observed a dramatic shift of TRIM32 localization from CBs to the diffuse cytoplasm fraction, clearly demonstrating that MB exposure disrupts CB structure (Fig 3B). This shift was initially detected within 2 h of MB exposure, and was observed in all TRIM32-positive cells after 4 h, with no significant difference among TRIM32 levels at each time point (Fig 3C). To determine whether the dispersed TRIM32 molecules in the MB-treated cells are soluble, we fractionated the cell extracts with low-speed centrifugation (9,000 × g for 10 min). Subsequent immunoblotting with anti-FLAG antibody revealed that most TRIM32 molecules were still present in the sediment fraction (Fig 3D), suggesting that the dispersed TRIM32s were insoluble and possibly present as oligomeric clusters. To further evaluate these findings using chemical compounds affecting HSP70 ATPase activity, we employed AlaC and SW02, which are respectively an HSP70-ATPase inhibitor and activator [36]. As expected, AlaC disrupted TRIM32-containing CBs similarly to MB, but SW02 did not (Fig 3E). Additionally, only 47% of the AlaC-treated, TRIM32-positive cells contained the remaining CBs, compared with 69% of non-treated cells (Fig 3F). We also verified that the number of cells with CBs significantly increased to >80% upon activation of endogenous HSP70 ATPase activity by SW02 (Fig 3F). Thus, these results suggest that a functional HSP70 chaperone is required for TRIM32-containing CBs not only to promote their assembly but also to maintain their structural integrity once they form in the cell. Consistent with the findings, we observed that knockdown of HSP70 by siRNA specifically suppressed the CB inclusion formation (S4 Fig).

A previous study showed that preexisting TRIM5-containing CBs are highly mobile and rapidly exchange protein components amongst themselves [9]. It has also been demonstrated that the cellular motility of TRIM aggregates is dependent on an intact microtubule network [8,9,18]. To exclude the possibility that our MB treatment also disrupted the microtubule network and that this disruption caused the observed CB phenotype indirectly, we compared MB-treated and non-treated HEK293 cells by microscopy after staining with an anti-tubulin monoclonal antibody. We confirmed that cells under both conditions indeed retained filamentous microtubule architectures and were morphologically indistinguishable from each other (S5 Fig). Thus, our findings regarding the disruption of CB structures in HEK293 cells appear to reflect a specific effect of MB on HSP70 ATPase activity.

### Active HSP70 chaperone also stabilizes TRIM63-containing CBs

TRIM63, also called MURF1 or RNF28, is a TRIM family member that contains the COS domain (C-terminal subgroup one signature) at the C-terminus [38]. Using the same assays performed for TRIM32, we looked for HSP70 binding and MB effects in TRIM63. Similar to our TRIM32 results, we found that endogenous HSP70 specifically bound to TRIM63 (Fig 4A). Additionally, MB reduced total TRIM63 levels (Fig 4B, lower panel) and disrupted the pre-existing CB structures in the cell (Fig 4C). Thus, we concluded that the HSP70 chaperone is also required for TRIM63-containing CBs to maintain structural stability in HEK293 cells.

### TRIM32 forms distinct complexes with HSP70 and 14-3-3

**Fig 5:**
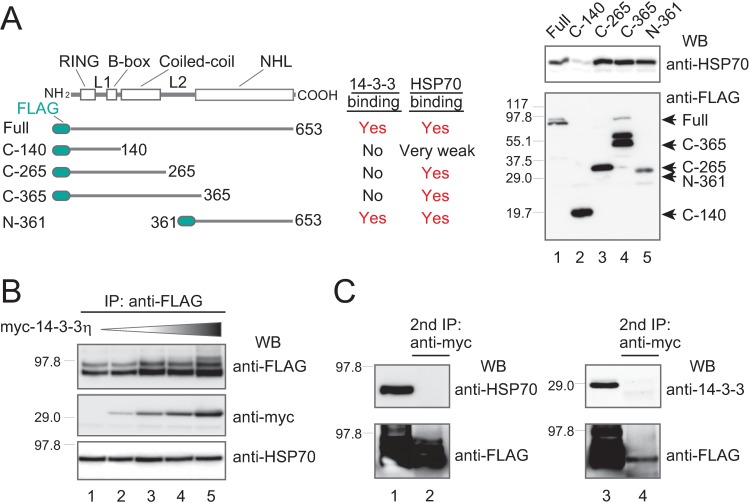
The HSP70-TRIM32 complex is biochemically separate from the 14-3-3-TRIM32 phospho-complex. (**A**) HEK293 cells were transiently transfected with FLAG-TRIM32 or its truncated mutants, immunoprecipitated with anti-FLAG, then western-blotted with anti-HSP70 and anti-FLAG. The 14-3-3 binding data are from our previous study [8]. “Full,” “C-140,” “C-265,” “C-365,” and “N-361” indicate the full TRIM32 protein and its truncated mutants. (**B**) HEK293 cells were transfected with FLAG-TRIM32, the PKA catalytic subunit, and increasing amounts of myc-14-3-3η (indicated by the shaded triangle above the western blot [WB]). Transfection was followed by immunoprecipitation with anti-FLAG, and then western blots, as described in (A). (**C**) The FLAG-TRIM32 complex shown in lane 5 of (B) was further subjected to immunoprecipitation with anti-myc Sepharose beads to purify the myc-14-3-3-FLAG-TRIM32 complex, and western-blotted with specific anti-HSP70 antibody to monitor endogenous HSP70 presence (left panel). The right panel shows the myc-HSP70-FLAG-TRIM32 complex with a western blot using anti-14-3-3 antibodies to monitor endogenous 14-3-3 presence.

Our previous work showed that 14-3-3s bind directly with TRIM32 when PKA phosphorylates the latter at Ser651 in the NHL domain. This association represses the formation of TRIM32-containing CBs by retaining the phosphorylated TRIM32 dimer in the cytosol [8]. Because 14-3-3 exerts an inhibitory effect on CB formation that is opposite of HSP70’s role, we determined whether these proteins formed a single TRIM32 complex, or two separate and distinct TRIM32 complexes. We thus compared the TRIM32 site(s) responsible for 14-3-3 and HSP70 binding by expressing four FLAG-tagged TRIM32 deletions (Fig 5A, left panel) in HEK293 cells. These deletions have truncated N- or C-terminals, and all four have been previously used to localize the 14-3-3 binding site ([8]; Fig 5A, middle panel). Subsequent immunoprecipitation with anti-FLAG antibody and western blotting with anti-HSP70 antibody revealed that endogenous HSP70 associated strongly with full-length TRIM32, as well as with the C-365, C-268, and the N-361 deletions, but not with the C-140 deletion (Fig 5A, right panel). These results suggest that the TRIM32 C-terminal NHL domain is a common binding site for HSP70 and 14-3-3, although other regions including the coiled-coil may associate with only HSP70.

We then transfected HEK293 with the PKA catalytic subunit, full-length FLAG-TRIM32, and increasing amounts of myc-14-3-3η (Fig 5B). Under PKA phosphorylation, the amount of FLAG-TRIM32 immunoprecipitates was dose-dependent on 14-3-3η expression (Fig 5B, top panel). This pattern can be explained by the enhanced solubility of the 14-3-3-FLAG-TRIM32 complex (Fig 5B, middle panel), as reported previously [8]. This complex does not appear to contain HSP70, as endogenous HSP70 levels in the TRIM32 immunoprecipitates remained unchanged under all tested conditions (Fig 5B, bottom panel).

To confirm HSP70 absence, we used anti-myc to immuno-purify the myc-14-3-3-TRIM32 complex from total FLAG-TRIM32 complexes. The resultant complexes were western-blotted with anti-HSP70 antibody, revealing no detectable HSP70 protein in the myc-14-3-3-TRIM32 complex (Fig 5C, left panel, lane 2). We also expressed myc-HSP70 instead of myc-14-3-3η and verified that the myc-HSP70-TRIM32 complex does not contain detectable levels of endogenous 14-3-3s (Fig 5C, right panel, lane 2). These results provided direct evidence that TRIM32 forms distinct, separate complexes with 14-3-3 and HSP70, in line with the observation that 14-3-3 interaction retains TRIM32 in the soluble cytosolic fraction, whereas HSP70 binding promotes TRIM32 accumulation into insoluble CB clusters. Although 14-3-3 and HSP70 are known to share many substrates, these results are the first direct comparison of their interactions with the same target molecule (TRIM32) in a common cell line (HEK293).

In the present study, we showed that HSP70 has a specific ATP-regulated interaction with overexpressed TRIM32, which enhances and stabilizes TRIM32-CB inclusions. These results support the notion that inclusion formation is actively controlled by cellular QC machinery, rather than merely being a failure of the QC process. Previous investigations corroborate our findings. For example, Zhang and Qian [39] have reported that HSP70 knockdown, in the presence of proteasome inhibitor MG132, prevents the aggregation of CHFP-VHL, a model JUNQ substrate (juxtanuclear quality control compartment; Kaganovich et al. [40]). Additionally, experiments using HSP70-deficient yeast strains have shown that the formation of Q-bodies is defective in HSP70Δ strains [41]. Furthermore, the presence of either the dominant-negative K71S mutant or an HSP70 inhibitor (VER155006) prevents the formation of cytoplasmic spots containing p53 R175H mutant [28]. Taken together, these data suggest that the HSP70 may function as a general promoter of aggregation, regardless of whether the aggregates are derived from physiological (e.g., TRIM32) or mutated proteins (e.g., p53 R175H).

**Fig 6:**
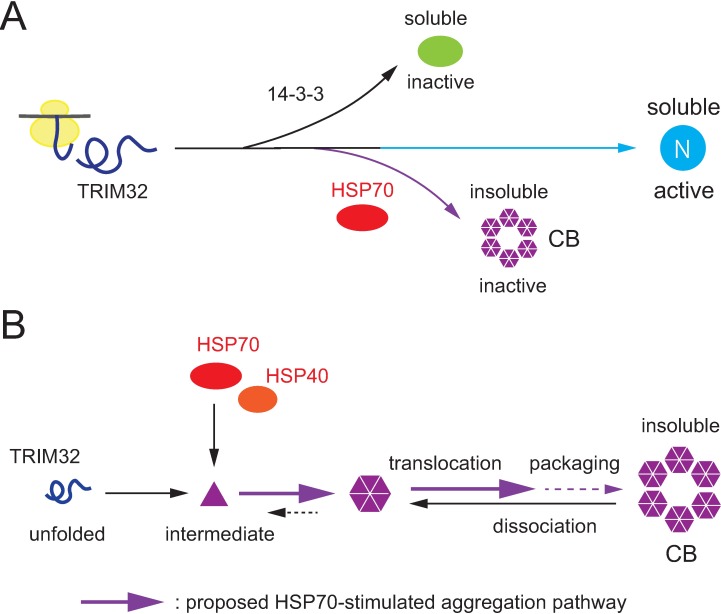
Schematic illustration of the proposed role for HSP70 in TRIM32-containing-CB formation. (**A**) Intracellular localization of TRIM32 is differentially controlled by 14-3-3 and HSP70. (**B**) HSP70 binds folding intermediates of TRIM32 and accelerates their accumulation into CBs.

Here, we suggest a model for the potential role of HSP70 chaperone in the CB assembly pathway (Fig 6). This model supposes the existence of at least two ATP-dependent systems controlling TRIM32 intracellular localization: 14-3-3-mediated retention in the soluble cytosolic fraction and HSP70-stimulated accumulation into insoluble CBs (Fig 6A). During de novo TRIM32 folding, any misfolded or partially folded intermediates are specifically bound by the HSP70 chaperone, which facilitates their localization into CBs in concert with the action of the co-chaperone HSP40 (Fig 6B). Once CBs form, the functional HSP70 chaperone also stabilizes their structure in cells (Fig 6B). This model is supported by our results including the demonstration that HSP70 is a major binding partner of overexpressed TRIM32 (Fig 1), that overexpression of HSP70 promotes the formation of TRIM32-containing CBs where this effect is further enhanced by co-expression of HSP40 (Fig 2), and that any removal of HSP70 ATPase activity (whether through mutants or pharmacological inhibitors) disrupts the CB structures, causing the formation of very small TRIM32 clusters dispersed in the cytoplasm (Figs 2 and 3). We propose that the HSP70 chaperone is a key player in the cellular machinery that mediates the active buildup and maintenance of TRIM32-containing CBs. Importantly, however, we were unable to detect any co-localization of HSP70 and CBs, at least at significant levels (see Fig 2A). This suggests that this chaperone could play a role before the final packaging of TRIM32 into CBs. Previous studies have shown that knockdown of BAG3 (BCL2-associated athanogene 3) caused the formation of punctured small aggregates that apparently failed to be packed into the juxtanuclear inclusion body in the presence of CHIP (carboxyl-terminus of Hsp70-interacting protein) [39]. It has also been reported that BAG3, similarly to other members of the BAG family including BAG1 and BAG2, acts as a nucleotide exchange factor for HSP70 and promotes the ATP-trigged release of substrates from the chaperone complex [42]. Because overexpression of the dominant-negative HSP70 K71S mutant exhibited a similar phenotype with small TRIM32 aggregates (Fig 2A–2D) and because this variant probably lacks the ability to release substrates, as it constantly bound to overexpressed TRIM32 polypeptides regardless of the presence or absence of ATP (Fig 1C), we assume that earlier dissociation of HSP70 from TRIM32 in an ATP-regulated reaction cycle might be an important step through which TRIM32 enters the subsequent packaging route. Further studies are needed to test this and other possibilities to define the detailed mechanisms underlying the role of HSP70 in CB formation. It is also important to clarify whether or how other QC elements, such as HSP104 [43] and small HSPs [44], work cooperatively with HSP70 in the CB assembly pathway.

In conclusion, our results demonstrate that CB inclusion formation is an energy-consuming reaction that is regulated by the HSP70 chaperone in cells. Thus, it appears to be part of normally functioning cellular QC, similar to protein folding and degradation. More specifically, the active control of CB formation may be a defense mechanism that sequesters potentially harmful overexpressed TRIM32 species from the cytosolic milieu. Because deregulated expression of TRIM32 contributes to various diseases, better understanding of the QC function and its relation to the disease process will be beneficial towards the design of new therapeutic approaches for TRIM32-associated diseases.

## Supporting Information

S1 Fig **Immunofluorescence images of TRIM32-containing CBs (A) and the turnover of expressed TRIM32 (B) in HEK293 cells.** (**A**) HEK293 cells were transiently transfected with FLAG-TRIM32, and TRIM32 localization was analyzed with immunofluorescence with monoclonal mouse anti-FLAG (green). (**B**) HEK293 cells were transiently transfected with FLAG-TRIM32 for 24 h and then incubated with cycloheximide (CHX; 100 μg/mL). Total proteins were extracted with 1% SDS at 0, 6, 12, and 18 h of incubation with CHX, and the turnover of FLAG-TRIM32 was analyzed using western blotting with anti-FLAG. Results were normalized to 1 at time 0 for each experiment and expressed as percentages.
(EPS)

S2 Fig HSP70 promotes CB assembly through associating with TRIM32. (A) HEK293 cells were transfected with FLAG-TRIM32 together with myc-HSP70 (wt) or myc-V438F (V438F). The lysates were separated with SDS-PAGE and western-blotted with the indicated antibodies (lower panels, marked as “Ext”). FLAG-TRIM32 proteins were also immunoprecipitated from the lysates with anti-FLAG, and the association between TRIM32 and HSP70 or the V438F mutant were analyzed through western blotting (upper panels, marked as “IP”). Note that the V438F mutant does not have the ability to associate with TRIM32 (upper panels, lane 2). (B) HEK293 cells were transfected with FLAG-TRIM32 and the myc-HSP70 constructs (names indicated in yellow), and the assembly of TRIM32-containing CBs was assessed according to Fig 2A. Note that, unlike wild-type HSP70, the co-expressed V438F cannot promote the CB assembly. Bars, 10 μm.
(EPS)

S3 Fig HSP90 does not affect the formation of TRIM32-containing CBs. (**A**) HEK293 cells were transiently transfected with FLAG-TRIM32 (+) or vector alone (-) together with myc-HSP90 or myc-D88N. The association between TRIM32 and HSP90 or the D88N mutant was analyzed with co-immunoprecipitation followed by western blotting. (**B–E**) CB characteristics and the number of CB-containing transfected HEK293 cells, as analyzed according to Fig 2A–2D. In (D), * indicates P = 0.24 and ** indicates P = 0.6. In (E), * indicates P = 0.10 and ** indicates P = 0.06. Bars, 10 μm.
(EPS)

S4 Fig HSP70 knockdown suppresses the formation of TRIM32-containing CBs. (A) HEK293 cells were transfected with control siRNA (lane 2) or HSP70 siRNA (lane 3) (10 μM each) or mock (lane 1, all reagents except for siRNA). The cell lysates were collected 96 h later and analyzed using western blotting with a monoclonal antibody specific to HSP70 (top panel). The intensity of each HSP70 band was quantified with a densitometer and expressed as a percentage of the intensity of the mock treatment (bottom panel). (B) HEK293 cells pre-incubated with control siRNA or HSP70 siRNA (10 μM each) for 70 h were transfected with FLAG-TRIM32, and incubation proceeded for further 26 h (total incubation time of 96 h post-siRNA transfection). Representative cell images from two independent experiments are shown. Bars, 10 μm. (C) The number of cells with CB inclusions were counted from 200 cells and expressed as a percentage as shown in Fig 3F. Note that HSP70 siRNA, but not control siRNA, specifically reduced the CB size and number inside cells (B) as well as the total number of cells that contained CBs (C).
(EPS)

S5 Fig Fluorescence image of the microtubule network treated with or without MB in HEK293 cells. HEK293 cells with preformed TRIM32 CBs were exposed to DMSO or MB for 4 h in the presence of CHX and stained with monoclonal mouse anti-tubulin. Representative images of cells from at least two independent experiments are shown. Note that MB exposure does not disrupt the intact microtubule network. Bars, 10 μm.
(EPS)

S1 Movie Three dimensional image of TRIM32-containing CBs in HEK293 cells. HEK293 cells were transfected with FLAG-TRIM32 and visualized using a DeltaVision microscope system according to S1A Fig. Three-dimensional image reconstruction was performed with the SoftWoRx software package.
(MPG)

S2 Movie Three dimensional image of TRIM32-containing CBs in HSP70-coexpressing HEK293 cells. HEK293 cells were transfected with FLAG-TRIM32 and myc-HSP70 and analyzed according to Fig 2B and S1 Movie. Note that myc-HSP70-expressing cells contain crowded CB clusters that may continue coalescing into larger CBs.
(MPG)
